# Research on UAV Autonomous Trajectory Planning Based on Prediction Information in Crowded Unknown Dynamic Environments

**DOI:** 10.3390/s25237343

**Published:** 2025-12-02

**Authors:** Jianing Tang, Songyan Yang, Shijie Chen, Qiao Li, Qian Yin, Sida Zhou

**Affiliations:** Yunnan Key Laboratory of Unmanned Autonomous Systems, School of Electrical and Information Engineering, Yunnan Minzu University, Kunming 650500, China; 041749@ymu.edu.cn (J.T.); yangsongy@ymu.edu.cn (S.Y.); 042178@ymu.edu.cn (S.C.); 24214038160025@ymu.edu.cn (Q.L.); cassie@ymu.edu.cn (Q.Y.)

**Keywords:** trajectory prediction, trajectory planning, autonomous vehicle navigation

## Abstract

When unmanned aerial vehicles (UAVs) operate autonomously in ultra-low-altitude environments, they encounter complex dynamic obstacles in the form of dense crowds. The high uncertainty and complex interactions in crowd movement pose significant challenges to the safe flight of UAVs. To address these issues, this paper proposes an integrated UAV trajectory planning method that combines pedestrian trajectory prediction with gradient-based planning. First, a Contrastive Distribution Latent Code Generator (CDLCG) is designed in the pedestrian trajectory prediction model to infer future trajectory distributions from pedestrians’ historical trajectories and generate predicted trajectories via a decoder. The accuracy and effectiveness of this method are validated using simulation methods based on four public datasets and validated through physical experiments on the OptiTrack Motion Capture System, respectively. Furthermore, an adaptive gradient-based UAV trajectory planning method is proposed by designing adaptive cost weights based on optimization stages and obstacle types. The method is validated in dynamic environments with varying crowd densities constructed in the Gazebo simulation environment, with results demonstrating that this method significantly improves the success rate of UAV trajectory planning in crowded dynamic environments; effectively balances trajectory smoothness, safety, and feasibility; and ensures safe UAV flight.

## 1. Introduction

With the growing demand for UAV applications, application scenarios are gradually transitioning from traditional high-altitude environments to complex low-altitude settings, particularly demonstrating significant potential in emerging fields such as urban logistics delivery and emergency search and rescue [1]. This transition presents unprecedented technical challenges. Compared to traditional high-altitude flight environments, dense pedestrian populations in low-altitude settings represent the most characteristic dynamic obstacles, whose motion trajectories exhibit significant multimodal uncertainty. The same initial state may correspond to multiple plausible future trajectories, a characteristic stemming from the randomness of pedestrian decision-making and complex social interactions [2]. This renders traditional deterministic prediction methods inadequate for effective handling. Meanwhile, the complexity of pedestrian movement significantly increases the difficulty of UAV trajectory planning, while the density of low-altitude environments further requires UAVs to possess rapid replanning capabilities, imposing higher computational efficiency requirements on methods. To address these challenges, UAVs require high-precision pedestrian trajectory prediction capabilities and real-time trajectory planning abilities.

In trajectory prediction, current mainstream prediction methods can be broadly classified into two categories: kinematic model-based prediction methods [3] and deep learning-based prediction methods [4,5]. The former solves for safe trajectories by establishing differential equations for obstacles. However, practical applications require precise calibration of numerous physical parameters such as mass distribution and air resistance coefficients, which not only leads to high model calibration costs but also makes performance heavily dependent on modeling accuracy, rendering adaptation to complex and variable real environments difficult [6]. The latter avoids the complexity of model construction and mainly employs autoregressive models based on Recurrent Neural Networks (RNNs) [7] and Transformer architectures [8] to progressively generate future trajectories. However, these methods suffer from significant error accumulation problems and inference efficiency bottlenecks [9], limiting their deployment in real environments.

Non-autoregressive methods that have emerged in recent years offer promising solutions to overcome this technical bottleneck. For example, the MRGTraj model [10] achieves parallel trajectory generation through an innovative Mapping-Refinement-Generation decoder structure and combines latent code sampling techniques to simultaneously output multiple socially plausible trajectories, achieving breakthrough progress in both prediction performance and computational efficiency. Nevertheless, such methods still need to address prediction performance degradation caused by information asymmetry between training and inference phases. Specifically, during training, models can simultaneously access complete historical trajectories and future ground truth trajectories, thereby learning the probability distributions of trajectories. However, during inference, models can only rely on limited historical data for prediction. This training-inference information asymmetry triggers trajectory probability distribution shift problems, leading to prediction performance degradation when models are deployed in real environments.

In trajectory planning, current methods can be broadly classified into three categories: search-based methods, sampling-based methods, and optimization-based methods. Search-based methods such as the Dijkstra and A* algorithms systematically search discretized state spaces to find optimal trajectories and can guarantee globally optimal solutions in known environments [11]. However, the computational complexity of search methods grows exponentially with dimensionality, making them more suitable for two-dimensional environments, with computational overhead increasing dramatically when facing three-dimensional or high-dimensional state spaces. Sampling-based methods such as RRT [12] and PRM [13] algorithms explore continuous state spaces through random sampling strategies, effectively mitigating the curse of dimensionality. However, their inherent randomness cannot guarantee solution optimality, and generated trajectories are often insufficiently smooth and lack kinodynamic constraints. Additionally, in dynamic environments, frequent resampling is required to handle obstacle state changes, incurring significant computational overhead. Given these challenges, collision avoidance emerges as a critical component in trajectory planning for UAV operating in complex and dynamic environments [14,15].

Alternatively, optimization-based methods formulate trajectory planning as mathematical optimization problems, solving directly in continuous parameter spaces by constructing objective functions containing multiple constraints. The core advantage of these methods lies in their ability to simultaneously optimize multiple performance metrics of trajectories, generating trajectories with superior execution quality. Among them, gradient-based methods utilize gradient information of objective functions to guide search directions, effectively handling complex constraints and enhancing obstacle avoidance capabilities, thus receiving widespread attention. However, in dynamic environments, frequent changes in obstacle states require algorithms to possess real-time replanning capabilities, while the high computational cost of gradient optimization processes makes it difficult to meet the real-time requirements of UAVs in low-altitude dynamic scenarios.

**Figure 1:**
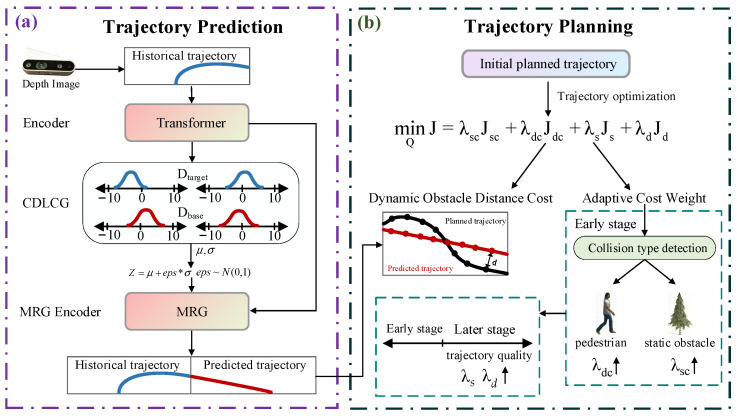
Overall Framework. (**a**) Trajectory Prediction. (**b**) Trajectory Planning.

Addressing the above challenges, this paper proposes an integrated UAV trajectory planning method that combines pedestrian trajectory prediction with gradient-based planning, achieving safe and efficient navigation of quadrotor UAVs in complex dynamic environments. As shown in Figure 1, the main contributions of this work are summarized as follows. The detailed implementations of both modules will be presented, respectively, in subsequent sections.

- This paper proposes a Contrastive Distribution Latent Code Generator (CDLCG), whose core innovation lies in guiding the predictive model to learn how to infer appropriate Gaussian distribution parameters from limited historical observation information in practical deployment scenarios. The model samples latent codes based on these distributions to produce a set of candidate prediction trajectories, and then selects optimal trajectories based on probabilistic evaluation.
- This paper proposes an integrated UAV trajectory planning method that combines pedestrian trajectory prediction with gradient-based planning. The method utilizes trajectory prediction techniques to obtain future motion trajectories of dynamic obstacles and integrates this prediction information into the trajectory planning process. Additionally, adaptive cost weights based on optimization stages and obstacle types are designed to achieve adaptive gradient-based trajectory planning.

## 2. Trajectory Prediction

In low-altitude complex environments where pedestrians serve as the primary dynamic obstacles, accurate prediction of pedestrian motion trajectories is crucial for ensuring safe UAV flight. To achieve this objective, this paper proposes a trajectory prediction method based on the existing non-autoregressive prediction model MRGTraj, which combines a Contrastive Distribution Latent Code Generator (CDLCG) with a non-autoregressive decoder.

As shown in Figure 1a, the prediction model comprises three core modules: an encoder, CDLCG, and MRG decoder. The encoder module utilizes a Transformer architecture to extract features from historical pedestrian trajectories. The CDLCG module represents the core innovation of this work. During the training phase, it learns how to infer reasonable Gaussian distributions from limited observational data through a contrastive learning mechanism, and generates latent codes from these distributions. These latent codes effectively capture and encode complex social interaction patterns within the environment. Subsequently, the MRG decoder module receives latent codes and historical trajectory features as inputs, performs parallel decoding to generate multiple reasonable future trajectory candidates, and selects superior trajectories based on probability. This method achieves reliable predictions using only limited historical observational information, providing more dependable trajectory prediction support for safe UAV navigation in crowded and complex dynamic environments.

### 2.1. Contrastive Distribution Latent Code Generator

In the process of predicting future pedestrian trajectories, the inherent randomness of individual movement patterns typically results in the existence of multiple socially plausible future trajectories. The core challenge in trajectory prediction lies in effectively modeling the uncertainty associated with motion. Existing models usually introduce latent codes to represent this uncertainty. Latent codes encode various potential future movement patterns based on historical observations. By sampling latent codes from reasonable Gaussian distributions, the model is capable of generating diverse and realistic future trajectories.

Traditional latent code generation methods typically adopt CVAE structures based on historical information or assume they follow Gaussian distributions, learning the corresponding Gaussian distribution parameters from both historical and future trajectories during the training phase. However, the former struggles to adequately capture complex social interaction patterns, while the latter suffers from a critical limitation. During the inference phase, since the true future trajectories are unknown, the model cannot infer appropriate Gaussian distribution parameters based on the social characteristics of the current environment and must resort to sampling latent codes from a standard Gaussian distribution. This inconsistency between training and inference phases leads to performance degradation when the model is deployed in practice. To address this limitation, this paper proposes a Contrastive Distribution Latent Code Generator that employs a dual distribution learning strategy, enabling the model to learn how to infer reasonable Gaussian distributions from limited information during the training phase. This approach ensures that even without future information during inference, the model can still generate high-quality predictions based on the learned distributions. The entire process mainly includes three steps: social feature extraction, dual distribution learning strategy, and latent code generation. This workflow is illustrated in Figure 1b.

### 2.2. Social Feature Extraction

In the first step, the model needs to extract key social features from both historical trajectories and future trajectories separately. Assume there are N pedestrians in the scene, given the historical trajectories of N pedestrians in the scene as $X=\left\{X_{1},X_{2},\ldots ,X_{N}\right\}$, where $X_{i}=\left\{p_{i}^{1},p_{i}^{2},\ldots ,p_{i}^{T}\right\}$ represents the position sequence of pedestrian i over the past T time steps. First, for pedestrian i at a specific time step t, the model calculates its basic state information, namely the current position $p_{i}^{t}$ and current velocity $v_{i}^{t}$. Next, the model computes the relative features $p_{ij}$, $v_{ij}$ and Euclidean distance $d_{ij}$ of each neighboring pedestrian j with respect to target pedestrian i, combining these features into $T_{ij}$:

(1) $$T_{ij}=[p_{ij},v_{ij},d_{ij}]$$

Considering that not all pedestrians will have an impact on the target pedestrian, we use a neighbor mask M to filter effective social relationships. If pedestrian j is a neighbor of pedestrian i within the influence distance threshold, then $M_{ij}=1$, otherwise $M_{ij}=0$. Finally, the model processes each neighbor’s feature information through a Multi-Layer Perceptron (MLP) and calculates all social features $C_{t}$ of pedestrian i at time step t through weighted aggregation, as shown in Equation (2):

(2) $$C_{t}=\sum_{j=1}^{N}M_{ij}MLP(T_{ij})$$

### 2.3. Dual Distribution Learning Strategy

In the second step, the model employs a dual distribution learning strategy to capture the uncertainty of future trajectories. The core idea of this strategy is that since future information is unavailable during inference, such information constraints should be emulated during training, while still leveraging complete information to provide supervisory signals. During the training phase, the model simultaneously computes two Gaussian distributions: the target distribution and the base distribution. The target distribution $p_{\mathrm{target}}$ utilizes pedestrian i’s historical trajectory H, social features $C_{t}$, and future trajectory F to learn distribution parameters, representing the ideal distribution when complete information is available:

(3) $$D_{\mathrm{target}}=N(\mu_{\mathrm{target}}(H,C_{t},F),\Sigma_{\mathrm{target}})$$

where $\mu_{\mathrm{target}}$ is the conditional mean parameter of the target distribution $D_{\mathrm{target}}$, and $\Sigma_{\mathrm{target}}$ is the conditional covariance parameter of the target distribution, usually assumed to be a diagonal matrix. The base distribution only uses historical trajectories and social features to learn distribution parameters, simulating the actual situation during inference:

(4) $$D_{base}=N(\mu_{base}(H,C_{t}),\Sigma_{base})$$

where $\mu_{\mathrm{base}}$ is the conditional mean parameter of the base distribution $D_{base}$, and $\Sigma_{base}$ is the conditional covariance parameter of the base distribution, usually assumed to be a diagonal matrix. To make the base distribution approximate the target distribution, we introduce Kullback–Leibler (KL) Divergence loss to measure the difference between the two distributions [16,17]:

(5) $$L=KL(D_{\mathrm{target}}||D_{base})$$

By minimizing the KL divergence between the two distributions, the model learns how to approximate the ideal distribution under information constraints, thereby enabling accurate predictions during the inference phase.

### 2.4. Latent Code Generation

The third step is to generate latent codes z from the learned distribution parameters [18]. To support diverse trajectory generation, we can sample K latent codes from the learned distribution:

(6) $$z=\mu_{base}+\sigma_{base}⊙\varepsilon ,\varepsilon ∼N(0,1)$$

where ⊙ denotes element-wise multiplication, and ε is Gaussian noise. The log probability corresponding to each sampled latent code is:

(7) $$\log p(z)=-\frac{1}{2}\sum_{j=1}^{d_{z}}\left[\frac{{\left(z_{j}-\mu_{basej}\right)}^{2}}{\sigma_{basej}^{2}}+\log(2\pi \sigma_{basej}^{2})\right]$$

Finally, the sampled latent codes and historical trajectory features are converted into actual predicted trajectories through the MRG decoder.

## 3. Trajectory Planning

This section proposes an integrated UAV trajectory planning method that combines pedestrian trajectory prediction with gradient-based planning. The method is based on the quadrotor local planning framework EGO-Planner [19] and includes two core modules: optimization problem construction and adaptive cost weight.

Through the synergistic interaction and collaborative optimization of these two modules, the method achieves a balance between efficiency and robustness, offering reliable technical support for trajectory planning in dynamic environments. As shown in Figure 1b, the optimization problem construction module formulates trajectory planning as a multi-objective optimization problem by integrating multiple cost components, including static obstacle distance cost, dynamic obstacle distance cost, smoothness cost, and feasibility cost. Notably, the dynamic obstacle cost incorporates the prediction outputs from the model described in Section 2. The adaptive cost weight module dynamically adjusts the weights of each cost function based on optimization stages and obstacle types. Specifically, the module emphasizes safety in early optimization stages and gradually shifts focus toward trajectory quality in later stages, while adopting different weight allocation strategies for static and dynamic obstacles.

### 3.1. Optimization Problem Formulation

To address the challenges posed by dynamic obstacles such as pedestrians or other risk factors, it is essential to ensure that UAVs are capable of performing rapid and efficient local optimization in order to effectively respond to uncertainties in unknown environments. The adoption of uniform B-spline trajectories represents a crucial step in achieving this objective. B-splines exhibit the local support property, which implies that modifying a single control point influences only the local geometry of the trajectory, without altering its global structure. This property makes B-splines particularly suitable for localized trajectory adjustment and optimization tasks. This paper uses the control point sequence $Q=\left\{Q_{0,}Q_{1,}\ldots Q_{n}\right\}$ as optimization variables, where $Q_{i}\in R^{3}$. The optimization problem can be formulated as the following optimization problem:

(8) $$\underset{Q}{\min}J=\lambda_{sc}J_{sc}+\lambda_{dc}J_{dc}+\lambda_{s}J_{s}+\lambda_{d}J_{d}$$

where $J_{sc}$, $J_{dc}$, $J_{s}$, and $J_{d}$ represent static obstacle distance cost, dynamic obstacle distance cost, smoothness cost, and feasibility cost, respectively, and $\lambda_{sc}$, $\lambda_{dc}$, $\lambda_{s}$, and $\lambda_{d}$ are the corresponding weight coefficients. The adjustment mechanism for these weights will be detailed in Section 3.2.

(1) Static Obstacle Distance Cost: The static obstacle distance cost is utilized to ensure that the trajectory’s control points maintain a safe distance from static obstacles. The construction method of the distance cost function is adopted from the referenced literature [19]. For each control point $Q_{i}$, the distance to the nearest obstacle j surface is defined as $d_{ij}$, and the cost function takes a piecewise form:
  (9) $$j_{sc}(i,j)=\left\{\begin{array}{ll} 0 & \;(s_{ij}\leq 0)\\ s_{ij}^{3} & \;(0<s_{ij}\leq s_{f})\\ 3s_{f}s_{ij}^{2}-3s_{f}^{2}s_{ij}+s_{f}^{3} & \;(s_{ij}>s_{f}) \end{array}\right.$$
  where $s_{f}$ is the safety distance threshold, and $s_{ij}=s_{f}-d_{ij}$. $j_{sc}(i,j)$ is the static obstacle distance cost generated by $Q_{i}$. When the control point is sufficiently far from obstacles ($d_{ij}$ > $s_{f}$), no penalty is incurred; when the control point is within the safety distance ($0<d_{i}\leq s_{f}$), the cost increases smoothly; when the control point enters the obstacle ($d_{i}\leq 0$), the cost increases rapidly. When multiple obstacles exist around a control point, the static obstacle distance cost generated by $Q_{i}$ is $j_{sc}(Q_{i})=\sum_{j=1}^{N_{p}}j_{sc}(i,j)$, where $N_{p}$ is the number of ${\left\{p,v\right\}}_{j}$ pairs for $Q_{i}$. The total static obstacle distance cost is:
  (10) $$J_{sc}=\sum_{i=1}^{n}j_{sc}(Q_{i})$$
  The gradient information for optimization can be obtained by taking the derivative of $J_{sc}$ with respect to $Q_{i}$ [20].

(2) Dynamic Obstacle Distance Cost: When pedestrians are detected, this paper integrates the predicted pedestrian trajectory information from the prediction model into local planning. This method not only accounts for the current positions of dynamic obstacles but also considers their potential future movement patterns, enabling proactive collision avoidance. Accordingly, a dynamic obstacle distance cost function is designed to incorporate the future motion trajectories of dynamic obstacles. First, the B-spline trajectory is divided into *M* consecutive time segments, with each segment storing all predicted positions of pedestrian j within that time interval. The *m*-th time segment corresponds to the time interval $\left[t_{m}^{start},t_{m}^{end}\right]$, where $t_{m}^{start}=m\Delta t$ and $t_{m}^{end}=(m+1)\Delta t$, with Δt being the time step. Second, the predicted trajectory of pedestrian j is temporally mapped to each time segment, constructing the segmented prediction set:
  (11) $$P_{j}^{m}=\left\{(t_{s},p_{j}(t_{s}))\left|t_{m}^{start}\leq t_{s}\leq t_{m}^{end}\right.\right\}$$
  which represents the collection of all predicted positions and time points of pedestrian j within the *m*-th time segment. Third, *K* time instants are uniformly sampled within the *m*-th time segment. For each sampled instant $t_{m}^{k}$, the UAV trajectory position is computed via the B-spline, and the corresponding pedestrian position is retrieved from the prediction set. If no prediction data exists at that instant, linear interpolation between adjacent predicted points is performed to ensure temporal continuity. Finally, the distance between the UAV and the pedestrian at instant $t_{m}^{k}$ is calculated as:
  (12) $$d_{j}(t_{m}^{k})=\left\|P_{\mathrm{UAV}}(t_{m}^{k})-P_{j}(t_{m}^{k})\right\|-r_{j}$$
  where $r_{j}$ is half the width of the pedestrian. The cost function takes a piecewise form:
  (13) $$j_{dck}(t_{m}^{k},j)=\left\{\begin{array}{ll} 0 & \;(s_{ijk}\leq 0)\\ s_{ijk}^{3} & \;(0<s_{ijk}\leq s_{f})\\ 3s_{f}s_{ijk}^{2}-3s_{f}^{2}s_{ijk}+s_{f}^{3} & \;(s_{ijk}>s_{f}) \end{array}\right.$$
  where $s_{f}$ is the safety distance threshold, and $s_{ijk}=s_{f}-d_{j}(t_{m}^{k})$. $j_{dck}(t_{m}^{k},j)$ is the dynamic obstacle distance cost generated by pedestrian j at instant $t_{m}^{k}$. When the UAV is sufficiently far from obstacles, no penalty is incurred; when the UAV is within the safety distance, the cost increases smoothly; when the UAV enters the obstacle, the cost increases rapidly. The total dynamic obstacle distance cost generated by pedestrian j across all time segments is:
  (14) $$j_{dc,j}=\sum_{m=1}^{M}\sum_{k=1}^{K}j_{dck}(t_{m}^{k},j)$$
  The aggregate dynamic obstacle distance cost for all pedestrians is:
  (15) $$J_{dc}=\sum_{j=1}^{N}$$
  where *N* is the number of detected pedestrians.

(3) Smoothness Cost: To ensure the physical feasibility of the predicted trajectory, we implement smoothness constraints by minimizing the high-order derivatives (acceleration) of the trajectory [21,22]. The smoothness cost is:
  (16) $$J_{s}=\sum_{i=1}^{n-1}{\left\|A_{i}\right\|}_{2}^{2}$$
  where $A_{i}$ is the acceleration of control point $Q_{i}$. The gradient for optimization can be obtained by taking the derivative of $J_{s}$ with respect to $Q_{i}$.

(4) Feasibility Cost: The construction method of the feasibility cost function is adopted from the referenced literature [19]. To ensure the trajectory conforms to kinematic constraints, the feasibility cost is:
  (17) $$J_{d}=\sum_{i=1}^{n}w_{v}F\left(V_{i}\right)+\sum_{i=1}^{n-1}w_{a}F\left(A_{i}\right)+\sum_{i=1}^{n-2}w_{j}F\left(J_{i}\right)$$
  where $w_{v}$, $w_{a}$, $w_{j}$, are the corresponding weight coefficients, and *F*(·) is a twice continuously differentiable metric function of higher order derivatives of control points.
  (18) $$F(C)=\sum_{r=x,y,z}f(c_{r})$$
  (19) $$f(c_{r})=\left\{\begin{array}{ll} a_{1}c_{r}^{2}+b_{1}c_{r}+c_{1} & \;(c_{r}\leq -c_{j})\\ {\left(-\lambda c_{m}-c_{r}\right)}^{3} & \;(-c_{j}<c_{r}<-\lambda c_{m})\\ 0 & \;(-\lambda c_{m}\leq c_{r}\leq \lambda c_{m})\\ {\left(c_{r}-\lambda c_{m}\right)}^{3} & \;(\lambda c_{m}<c_{r}<c_{j})\\ a_{2}c_{r}^{2}+b_{2}c_{r}+c_{2} & \;(c_{r}\geq c_{j}) \end{array}\right.$$
  where $c_{r}\in C\in \left\{V_{i},A_{i},J_{i}\right\}$, a1,b1,c1,a2,b2,c2 are chosen to meet the second-order continuity, $c_{m}$ is the derivative limit, $c_{j}$ is the splitting points of the quadratic interval and the cubic interval λ<1−ε is an elastic coefficient with 0<ε≪1 to make the final results meet the constraints, since the cost function is a tradeoff of all weighted terms [19]. The gradient for optimization can be obtained by taking the derivative of $J_{d}$ with respect to $Q_{i}$.

### 3.2. Adaptive Cost Weight

In gradient-based trajectory planning methods, traditional approaches typically employ fixed weight strategies, maintaining constant weights for each objective function throughout the entire optimization process. However, in complex dynamic environments, such methods exhibit notable limitations. Different optimization stages impose distinct requirements on each objective. The initial stage prioritizes the rapid identification of feasible, obstacle-avoiding solutions, whereas later stages emphasize trajectory quality. Fixed weights struggle to simultaneously satisfy these stage-specific requirements, easily leading to optimization getting trapped in local optima or slow convergence. Based on the above issues, this paper proposes an adaptive cost weight that dynamically adjusts corresponding weights based on optimization stages and obstacle types. This achieves more intelligent and efficient trajectory planning. First, we define the optimization progress indicator:

(20) $$\rho =\frac{k}{K},\rho \in \left[0,1\right]$$

where k is the current iteration number and K is the maximum number of iterations for the optimizer. Based on ρ, we divide the optimization process into two stages and design corresponding weight adjustment strategies for each stage. The weight adjustment rules are defined as:

(21) $$\lambda_{i}(\rho )=\lambda_{i}^{\left(0\right)}\cdot a_{i}(\rho )$$

where $i\in \left\{sc,dc,s,d\right\}$. $\lambda_{i}^{\left(0\right)}$ are the initial weights, and $a_{i}(\rho )$ is the adjustment function. The adjustment function is designed such that in the early stage of optimization when 0≤ρ<0.3, the static and dynamic cost weights are increased while smoothness and feasibility cost weights are reduced, enabling the optimizer to quickly find an initial feasible trajectory. After obtaining a basic feasible solution, the optimization focus shifts to balanced optimization of all objectives, increasing the weights for smoothness and feasibility.

Throughout the optimization iterations, collision detection is conducted every 50 iterations to assess whether the current trajectory is collision-free. If a collision is detected, the type of collision is identified. In the case of a dynamic obstacle collision, the corresponding dynamic obstacle distance cost weight is increased, otherwise, the static obstacle distance cost weight is increased. This adaptive cost weights method effectively balances trajectory smoothness, safety, and feasibility, thereby enabling efficient and robust trajectory planning.

## 4. Experiment Results

Two parts of experiments are carried out to evaluate the performance of the proposed method. The first part involves trajectory prediction error evaluation experiments, which assesses the accuracy of the prediction model in forecasting future trajectories to validate the method’s effectiveness. The second part consists of trajectory planning experiments, which evaluate the success rate of UAV trajectory planning under varying dynamic obstacle density scenarios to confirm the method’s reliability.

### 4.1. Trajectory Prediction and Error Evaluation

#### 4.1.1. Simulation Experiments of Trajectory Prediction

To systematically evaluate the generalization performance of the proposed model, we conducted comparative experiments against MRGTraj, which represents the current state-of-the-art non-autoregressive trajectory prediction model, across four representative public datasets. These four datasets comprise HOTEL, UNIV, ZARA1, and ZARA2 [23,24]. The HOTEL dataset was collected in hotel scenarios, the UNIV dataset was sourced from university campus environments, and both ZARA1 and ZARA2 were recorded in commercial street scenes. All experiments were conducted under the condition that no future trajectory information was provided during the inference phase.

For model evaluation, we employed the widely used Average Displacement Error (ADE) and Final Displacement Error (FDE) as evaluation metrics. ADE represents the average Euclidean distance d between all corresponding points of the predicted trajectory and the ground-truth trajectory, calculated as

(22) $$d=\frac{1}{T_{pred}}\sum_{t=1}^{T_{pred}}\sqrt{{\left(x_{pt}-x_{gt}\right)}^{2}+{\left(y_{pt}-y_{gt}\right)}^{2}}$$

where $T_{pred}$ is prediction time steps, $\left(x_{pi},y_{pi}\right)$ is predicted position at time step *t*, $\left(x_{gt},y_{gt}\right)$ is ground-truth position at time step *t*. while FDE denotes the Euclidean distance $d_{end}$ between the predicted endpoint $\left(x_{pend},y_{pend}\right)$ and the ground-truth endpoint $\left(x_{gend},y_{gend}\right)$, formulated as

(23) $$d_{end}=\sqrt{{\left(x_{pend}-x_{gend}\right)}^{2}+{\left(y_{pend}-y_{gend}\right)}^{2}}$$

**Figure 2:**
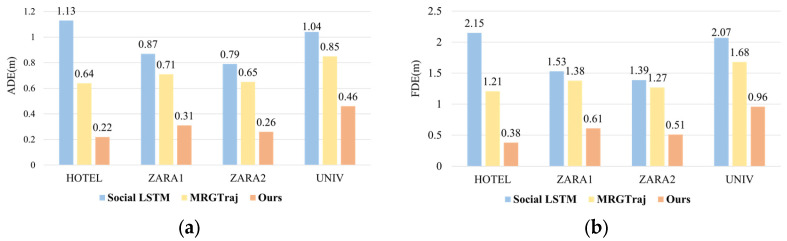
Trajectory prediction error results. (**a**) Average Displacement Error (ADE) and (**b**) Final Displacement Error (FDE).

To ensure statistical reliability of the experimental results, each experimental setting was independently repeated 20 times. For each trial, the error metrics were calculated based on the method’s final trajectory prediction. The experimental results are shown in Figure 2, where all numerical values represent the arithmetic mean of results obtained from 20 independent experiments conducted on each of the four datasets, with precision uniformly maintained to two decimal places.

Based on 20 repeated testing experiments, the average test results for the four scenarios are as follows: HOTEL: 0.22/0.38, ZARA1: 0.31/0.61, ZARA2: 0.26/0.51, UNIV: 0.46/0.96, all ADE values below 0.46 and all FDE values below 0.96. In comparison, the MRGTraj model on the same datasets exhibits ADE values exceeding 0.60 and FDE values greater than 1.2; the Social LSTM model shows ADE values exceeding 0.79 and FDE values greater than 1.39. Through analysis and calculations, compared with MRGTraj, the proposed model reduces prediction errors by 65%, 56%, 60%, and 45% for ADE and 68%, 55%, 59%, and 42% for FDE on the HOTEL, ZARA1, ZARA2, and UNIV datasets, respectively. Compared with Social LSTM, the proposed model reduces prediction errors by 80%, 64%, 67%, and 55% for ADE and 82%, 60%, 63%, and 53% for FDE on the HOTEL, ZARA1, ZARA2, and UNIV datasets, respectively. These results conclusively validate that the proposed model achieves significant performance improvements in trajectory prediction accuracy, demonstrating the superior reliability and precision of the proposed approach.

#### 4.1.2. Physical Experiments of Trajectory Prediction

To evaluate the performance of the proposed prediction model furthermore, physical experiments were conducted with a motion capture system under the real-world dynamic conditions. System configuration parameters are shown in Table 1.

**Figure 3:**
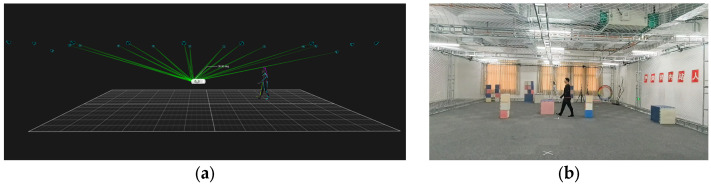
This figure illustrates a professional motion capture system setup, which consists of two main components. (**a**) the software interface and (**b**) the physical experimental space.

A professional motion capture system setup is shown in Figure 3. In Figure 3a, the system deploys 20 infrared cameras to establish a 360° full-coverage motion capture network within the experimental area. Participants wear specialized motion capture suits with spherical markers attached to key body parts. The system calculates the coordinates of the markers within the fields of view of each camera in real-time, enabling high-precision capture of human motion trajectories. Figure 3b shows the corresponding real-world experimental scenario, where a participant wearing a black motion capture suit walks within the experimental area, taking part in a pedestrian trajectory prediction experiment.

The experimental design follows a progressive validation strategy from simple to complex, including four representative walking patterns: straight walking, turning, circular walking, and two-person interactive walking. Straight walking involves participants walking along a predetermined linear trajectory to verify the model’s prediction accuracy under linear motion patterns. In the turning condition, participants make arbitrary turns during walking to test the model’s sensitivity and adaptability to directional changes. The circular-walking condition requires participants to walk along a circular trajectory, assessing the model’s long-term prediction stability under nonlinear motion. In the two-person interaction condition, two participants walk simultaneously in the same area, approaching and crossing each other, to test the model’s prediction performance in handling multi-pedestrian social interaction scenarios. In the data processing and evaluation phase, the trajectory data acquired by the motion capture system are utilized as ground truth trajectories and are compared with those generated by the prediction model. The evaluation metrics are defined as ADE and FDE, which serve as the primary performance indicators.

**Figure 4:**
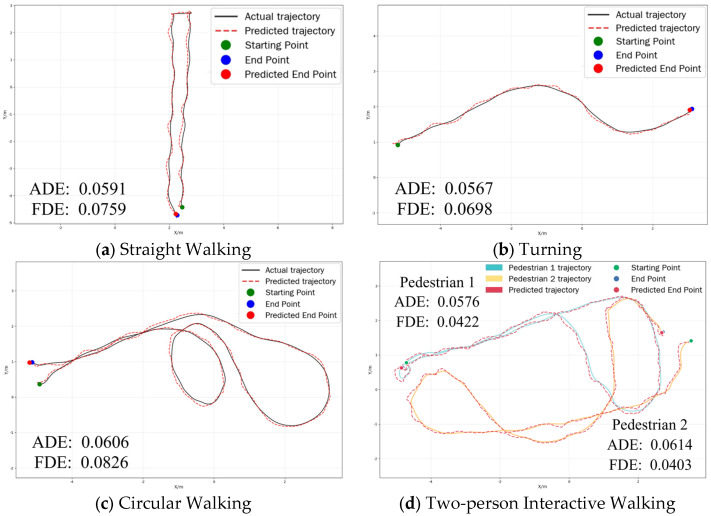
Visualization of the trajectory prediction results produced by the proposed model under various motion patterns.

As shown in Figure 4, the test results for the four walking patterns are as follows: Straight Walking: 0.0591/0.0759, Turning: 0.0567/0.0698, Circular Walking: 0.0606/0.0826, Two-person Interactive Walking: 0.0576/0.0422 (Pedestrian 1) and 0.0614/0.0403 (Pedestrian 2), representing the individual ADE/FDE for each pedestrian. The experimental results demonstrate that the proposed model maintains both ADE and FDE below 0.09 m across all test scenarios, that is merely 0.1% of the entire motion-capture area. Such low prediction errors can provide UAVs with reliable trajectory prediction results, thereby enabling enhanced proactive path planning capabilities in complex dynamic environments and ultimately achieving safer and more efficient autonomous navigation.

### 4.2. Trajectory Planning Experiments

**Figure 5:**
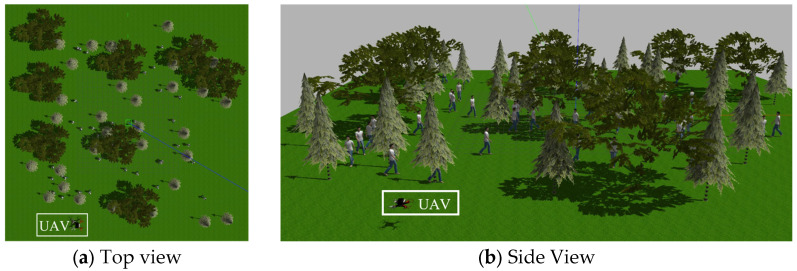
Simulation environment. (**a**) Top view and (**b**) Side view.

To verify the effectiveness of the proposed trajectory planning method, we constructed complex forest scenarios in the Gazebo simulation environment. The top and side views of the forest scenario are shown in Figure 5. The experiments were conducted on Ubuntu 20.04. The planning method was implemented in C++ within the ROS framework, and its 3-D performance was visualized in real time through Rviz.

The experiments utilized the simulation parameters specified in Table 2, which included forest scenarios featuring randomly distributed trees as static obstacles and variable numbers of dynamic pedestrian obstacles to emulate real-world environmental conditions. Pedestrians motion model employs a waypoint-based reciprocating pattern, where each pedestrian moves back and forth in straight lines between predefined start and end points, with walking speeds of v∈[0.5,1.0] m/s and angular velocities of ω∈[0.5,1.0] rad/s. While simplifying the complexity of social behavior modeling, this model retains the core uncertainty characteristics of pedestrian motion, particularly the dynamic changes in movement direction, thereby providing representative test scenarios for trajectory planning algorithms.

**Figure 6:**
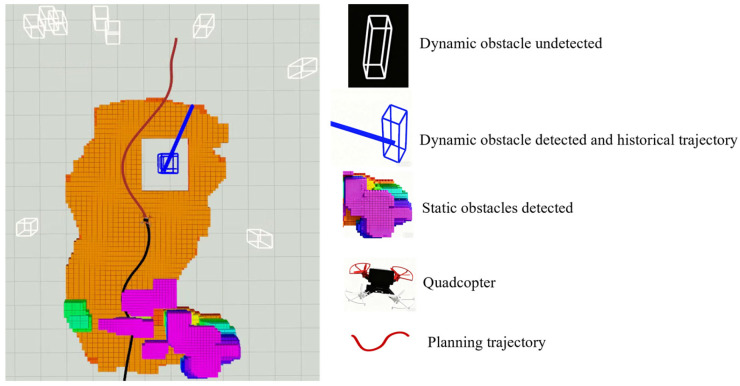
Trajectory planning process visualization in RVIZ.

Four comparative experiments were designed to compare the proposed method with the classical CERLAB [25] and EGO-Planner method. Both methods employed identical parameter configurations. In each experiment, the number of static obstacles remained constant while the number of dynamic obstacles was set to 5, 25, 50 and 80, respectively, to test obstacle avoidance performance under different densities. Figure 6 visualizes the trajectory planning process in Rviz. The gray grid areas represent unexplored regions; orange areas indicate explored safe flight zones without obstacles; other colors represent regions occupied by obstacles; white bounding boxes represent all dynamic obstacles undetected in the map; blue bounding boxes and blue trajectories represent dynamic obstacles detected during UAV’s flight and their historical trajectories, respectively; The red trajectory indicates the route currently being planned by the UAV, while the black trajectory represents its historical flight path.

**Figure 7:**
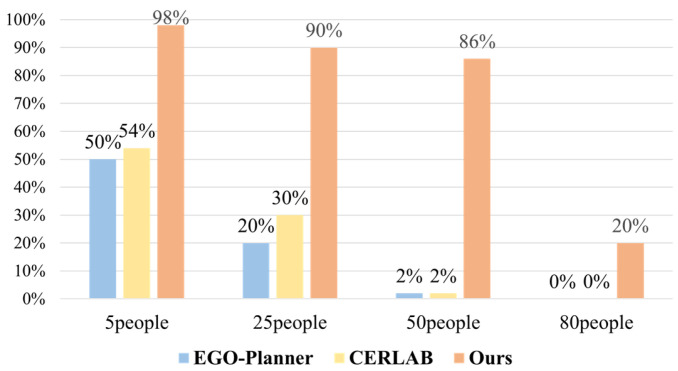
Trajectory planning success rate.

Each experiment was independently repeated 50 times, recording the number of successful collision-free arrivals at the target in each trial. The average of the 50 experimental results was used as the core metric for evaluating the method’s performance. The trajectory planning experiment success rates are shown in Figure 7. Compared with the CERLAB and EGO-Planner method, the proposed method achieves significant improvements in planning success rates across different density environments. In the 5-pedestrian scenario, the proposed method achieves a success rate of 98%, while EGO-Planner and CERLAB achieve 50% and 54%, respectively; in the 25-pedestrian scenario, the proposed method achieves a success rate of 90%, outperforming EGO-Planner (20%) and CERLAB (26%); in the 50-pedestrian scenario, the proposed method maintains an 86% success rate, while both baseline methods achieve only 2%; in the extremely dense 80-pedestrian scenario, the proposed method still achieves a 20% success rate, while both baseline methods fail completely (0%). These results fully demonstrate the robustness of the proposed method in complex dynamic environments with varying pedestrian densities.

To validate the effectiveness of the proposed adaptive weighting algorithm, we conducted a comparative analysis between our method and the baseline method CERLAB under identical parameter configurations and simulation environments, focusing on two key metrics, single-iteration optimization time and single-iteration total planning time. As shown in Table 3, The experimental results demonstrate that our proposed method significantly outperforms the CERLAB baseline in both optimization time and planning time, with the optimization time reduced from 1.6 ms to 1 ms and the planning time reduced from 1.9 ms to 1.2 ms. It is worth emphasizing that these computational efficiency gains are achieved while maintaining a high flight success rate, which fully validates the effectiveness of our proposed method.

## 5. Conclusions

This paper proposes an integrated trajectory planning method that combines pedestrian trajectory prediction with gradient-based planning, aiming to enhance the autonomous flight capability of UAVs in ultra-low-altitude environments with dense pedestrians. The method effectively improves pedestrian trajectory prediction accuracy by proposing a Contrastive Distribution Latent Code Generator (CDLCG), while enhancing the trajectory planning success rate of UAVs in dynamic environments through an adaptive gradient-based planning method. To validate the accuracy and effectiveness of the proposed method, we conduct trajectory prediction experiments on four public datasets (HOTEL, ZARA1, ZARA2, and UNIV) and an OptiTrack motion capture system. Compared with MRGTraj, the proposed model reduces prediction errors by 65%, 56%, 60%, and 45% for ADE and 68%, 55%, 59%, and 42% for FDE on the HOTEL, ZARA1, ZARA2, and UNIV datasets, respectively. Compared with Social LSTM, the proposed model reduces prediction errors by 80%, 64%, 67%, and 55% for ADE and 82%, 60%, 63%, and 53% for FDE on the HOTEL, ZARA1, ZARA2, and UNIV datasets, respectively. Meanwhile, dynamic environments with various pedestrian densities are constructed in the Gazebo simulation environment to test the UAV trajectory planning success rate. Compared with the classical CERLAB and EGO-Planner method, the proposed method achieves significant improvements in planning success rates across different density environments. These results fully demonstrate the robustness of the proposed method in complex dynamic environments with varying pedestrian densities. Experimental results demonstrate that the proposed method exhibits excellent performance in both pedestrian trajectory prediction and UAV trajectory planning, particularly showing high safety and robustness in dynamic environments with high pedestrian density.

## Figures and Tables

**Table 1:** System Parameter Configuration.

Parameter	Value
Motion Capture System	OptiTrack
Software	Motive
Number of Cameras	20
Camera Frame Rate	150 fps
Export Format	Csv
Exported Data Frame Rate	1000 fps
Dynamic capture accuracy	Sub-millimeter level

**Table 2:** Simulation Parameter Configuration.

Parameter	Value
Sensor perception range	9 m
Maximum flight speed	2 (m·s^−1^)
Maximum flight acceleration	3 (m·s^−2^)
Safety distance threshold	0.5 m
Map resolution	0.1 m
Map size	40 × 40 × 3 m
Number of pedestrian obstacles	5/25/50
Max pedestrian speed	1 (m·s^−1^)

**Table 3:** Planners Time Comparison.

Planner	$t_{opti}$	$t_{Plan}$
CERLAB	1.6 ms	1.9 ms
Ours	1 ms	1.2 ms

## Data Availability

The data that support the findings of this study are available from the corresponding author upon reasonable request.

## References

[1] Nikolic J., Burri M., Rehder J., Leutenegger S., Huerzeler C., Siegwart R. (2013). A UAV System for Inspection of Industrial Facilities. Proceedings of the 2013 IEEE Aerospace Conference.

[2] Alahi A., Goel K., Ramanathan V., Robicquet A., Fei-Fei L., Savarese S. Social Lstm: Human Trajectory Prediction in Crowded Spaces. Proceedings of the IEEE Conference on Computer Vision and Pattern Recognition.

[3] Truong X.-T., Ngo T.D. (2017). Toward Socially Aware Robot Navigation in Dynamic and Crowded Environments: A Proactive Social Motion Model. IEEE Trans. Autom. Sci. Eng..

[4] Truong X.-T., Ngo T.-D. (2017). “To Approach Humans?”: A Unified Framework for Approaching Pose Prediction and Socially Aware Robot Navigation. IEEE Trans. Cogn. Dev. Syst..

[5] Camara F., Bellotto N., Cosar S., Weber F., Nathanael D., Althoff M., Wu J., Ruenz J., Dietrich A., Markkula G. (2020). Pedestrian Models for Autonomous Driving Part II: High-Level Models of Human Behavior. IEEE Trans. Intell. Transp. Syst..

[6] Ammoun S., Nashashibi F. (2009). Real Time Trajectory Prediction for Collision Risk Estimation between Vehicles. Proceedings of the 2009 IEEE 5th International Conference on Intelligent Computer Communication and Processing.

[7] Zhang P., Ouyang W., Zhang P., Xue J., Zheng N. Sr-Lstm: State Refinement for Lstm towards Pedestrian Trajectory Prediction. Proceedings of the IEEE/CVF Conference on Computer Vision and Pattern Recognition.

[8] Yu C., Ma X., Ren J., Zhao H., Yi S., Vedaldi A., Bischof H., Brox T., Frahm J.-M. (2020). Spatio-Temporal Graph Transformer Networks for Pedestrian Trajectory Prediction. Computer Vision—ECCV 2020.

[9] Mohamed A., Qian K., Elhoseiny M., Claudel C. Social-Stgcnn: A Social Spatio-Temporal Graph Convolutional Neural Network for Human Trajectory Prediction. Proceedings of the IEEE/CVF Conference on Computer Vision and Pattern Recognition.

[10] Peng Y., Zhang G., Shi J., Li X., Zheng L. (2023). MRGTraj: A Novel Non-Autoregressive Approach for Human Trajectory Prediction. IEEE Trans. Circuits Syst. Video Technol..

[11] Hart P.E., Nilsson N.J., Raphael B. (1968). A Formal Basis for the Heuristic Determination of Minimum Cost Paths. IEEE Trans. Syst. Sci. Cybern..

[12] LaValle S.M. (1998). Rapidly-Exploring Random Trees: A New Tool for Path Planning.

[13] Hornung A., Wurm K.M., Bennewitz M., Stachniss C., Burgard W. (2013). OctoMap: An Efficient Probabilistic 3D Mapping Framework Based on Octrees. Auton. Robots.

[14] Singh A., Singh J., Sujit P.B. (2025). PANDA: Priority-Based Collision Avoidance Framework for Heterogeneous UAVs Navigating in Dense Airspace. Proceedings of the 24th International Conference on Autonomous Agents and Multiagent Systems.

[15] Huang S., Teo R.S.H., Tan K.K. (2019). Collision Avoidance of Multi Unmanned Aerial Vehicles: A Review. Annu. Rev. Control.

[16] Hershey J.R., Olsen P.A. (2007). Approximating the Kullback Leibler Divergence between Gaussian Mixture Models. Proceedings of the 2007 IEEE International Conference on Acoustics, Speech and Signal Processing-ICASSP’07.

[17] Cui J., Tian Z., Zhong Z., Qi X., Yu B., Zhang H. (2024). Decoupled Kullback-Leibler Divergence Loss. Adv. Neural Inf. Process. Syst..

[18] Higgins I., Matthey L., Pal A., Burgess C., Glorot X., Botvinick M., Mohamed S., Lerchner A. Beta-Vae: Learning Basic Visual Concepts with a Constrained Variational Framework. Proceedings of the International Conference on Learning Representations.

[19] Zhou X., Wang Z., Ye H., Xu C., Gao F. (2020). Ego-Planner: An Esdf-Free Gradient-Based Local Planner for Quadrotors. IEEE Robot. Autom. Lett..

[20] Yu Q., Qin C., Luo L., Liu H.H.-T., Hu S. (2022). Cpa-Planner: Motion Planner with Complete Perception Awareness for Sensing-Limited Quadrotors. IEEE Robot. Autom. Lett..

[21] Usenko V., Von Stumberg L., Pangercic A., Cremers D. (2017). Real-Time Trajectory Replanning for MAVs Using Uniform B-Splines and a 3D Circular Buffer. Proceedings of the 2017 IEEE/RSJ International Conference on Intelligent Robots and Systems (IROS).

[22] Zhou B., Gao F., Wang L., Liu C., Shen S. (2019). Robust and Efficient Quadrotor Trajectory Generation for Fast Autonomous Flight. IEEE Robot. Autom. Lett..

[23] Pellegrini S., Ess A., Schindler K., Van Gool L. (2009). You’ll Never Walk Alone: Modeling Social Behavior for Multi-Target Tracking. Proceedings of the 2009 IEEE 12th International Conference on Computer Vision.

[24] Lerner A., Chrysanthou Y., Lischinski D. (2007). Crowds by Example. Comput. Graph. Forum.

[25] Xu Z., Zhan X., Chen B., Xiu Y., Yang C., Shimada K. (2023). A Real-Time Dynamic Obstacle Tracking and Mapping System for UAV Navigation and Collision Avoidance with an RGB-D Camera. Proceedings of the 2023 IEEE International Conference on Robotics and Automation (ICRA).

